# Succinyl-CoA:acetate CoA-transferase functioning in the oxidative tricarboxylic acid cycle in *Desulfurella acetivorans*

**DOI:** 10.3389/fmicb.2022.1080142

**Published:** 2022-12-07

**Authors:** Eugenio Pettinato, Pauline Böhnert, Ivan A. Berg

**Affiliations:** Institute for Molecular Microbiology and Biotechnology, University of Münster, Münster, Germany

**Keywords:** CoA-transferases, tricarboxylic acid cycle (TCA cycle), acetate oxidation, *Desulfurella*, succinyl-CoA synthetase, acetyl-CoA synthetase

## Abstract

*Desulfurella acetivorans* is a strictly anaerobic sulfur-reducing deltaproteobacterium that possesses a very dynamic metabolism with the ability to revert the citrate synthase version of the tricarboxylic acid (TCA) cycle for autotrophic growth (reversed oxidative TCA cycle) or to use it for acetate oxidation (oxidative TCA cycle). Here we show that for heterotrophic growth on acetate *D. acetivorans* uses a modified oxidative TCA cycle that was first discovered in acetate-oxidizing sulfate reducers in which a succinyl-CoA:acetate CoA-transferase catalyzes the conversion of succinyl-CoA to succinate, coupled with the activation of acetate to acetyl-CoA. We identified the corresponding enzyme in this bacterium as the AHF96498 gene product and characterized it biochemically. Our phylogenetic analysis of CoA-transferases revealed that the CoA-transferase variant of the oxidative TCA cycle has convergently evolved several times in different bacteria. Its functioning is especially important for anaerobes, as it helps to increase the energetic efficiency of the pathway by using one enzyme for two enzymatic reactions and by allowing to spend just one ATP equivalent for acetate activation.

## Introduction

**FIGURE 1:**
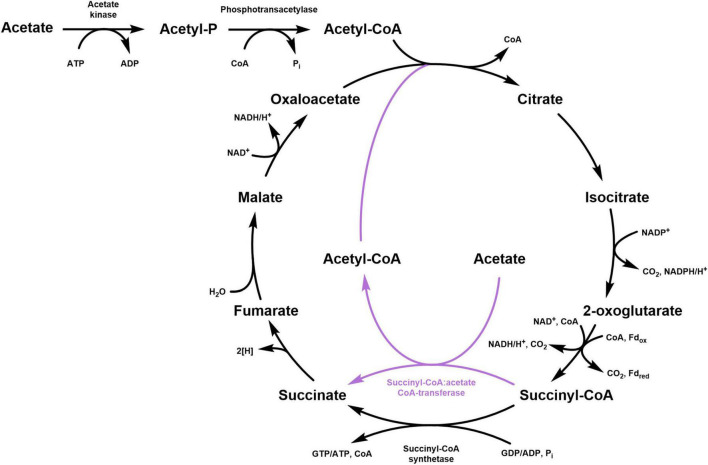
Schematic representation of the classical TCA cycle (black) and its modification involving a succinyl:CoA:acetate CoA-transferase (purple). For the 2-oxoglutarate decarboxylation, both NAD-dependent 2-oxoglutarate dehydrogenase (aerobic and facultative anaerobic organisms) and 2-oxoglutarate:ferredoxin oxidoreductase (strict anaerobes) are shown.

The tricarboxylic acid (TCA) cycle is the central metabolic pathway responsible for the oxidation of acetyl-CoA into two molecules of CO_2_ with the concomitant reduction of NAD(P) and quinone (Figure 1). This cycle functions in both aerobic and anaerobic respiration and starts with the condensation of oxaloacetate and acetyl-CoA to form citrate. Aconitase converts the tertiary alcohol citrate into the secondary alcohol isocitrate, thus allowing its further oxidation in the NAD(P)-dependent reaction catalyzed by isocitrate dehydrogenase that leads to the formation of 2-oxoglutarate and CO_2_. The oxidative decarboxylation of 2-oxoglutarate to succinyl-CoA is usually catalyzed by NAD-dependent 2-oxoglutarate dehydrogenase (in aerobes and facultative anaerobes) or by 2-oxoglutarate:ferredoxin oxidoreductase (in strictly anaerobic organisms). The energy of the thioester bond of succinyl-CoA is used by succinyl-CoA synthetase to form succinate and ATP or GTP. This is the only substrate-level phosphorylation step in the TCA cycle. Then, the formed succinate is oxidized by succinate dehydrogenase to fumarate while the electrons are transferred to (ubi) quinone in the cytoplasmic membrane. Lastly, fumarate is hydrated to malate, which is oxidized by malate dehydrogenase to oxaloacetate in a reaction that is usually NAD-dependent. The regeneration of oxaloacetate, the acceptor of acetyl-CoA in the citrate synthase reaction, closes the cycle.

Although this “classical” version of the TCA cycle is widespread, many variants have been described. For example, *Propionibacterium freudenreichii* converts 2-oxoglutarate to succinate *via* glutamate, 4-aminobutyrate and succinic semialdehyde (Beck and Schink, 1995), cyanobacteria perform this conversion with 2-oxoglutarate decarboxylase and succinic semialdehyde dehydrogenase (Zhang and Bryant, 2011), whereas aerobic halobacteria use ferredoxin-dependent 2-oxoglutarate oxidoreductase (Kerscher and Oesterhelt, 1981). Furthermore, some microorganisms use a quinone-dependent malate dehydrogenase (Molenaar et al., 2000; Harold et al., 2022) that is exergonic in the direction of malate oxidation, in contrast to the NAD-dependent enzyme. In addition, the ferredoxin-dependent TCA cycle can be reversed and used for autotrophic CO_2_ fixation (Evans et al., 1966; Berg, 2011; Fuchs, 2011; Buchanan et al., 2017). In this reductive version of the cycle, the citrate cleavage can be catalyzed by ATP-dependent citrate lyase (Ivanovsky et al., 1980), or by homologous citryl-CoA synthetase/citryl-CoA lyase (Aoshima et al., 2004a,b), or by citrate synthase in the reversed oxidative TCA cycle (Mall et al., 2018; Nunoura et al., 2018; Steffens et al., 2021). Interestingly, some bacteria use this pathway in the oxidative and reductive direction, depending on the growth conditions (Brandis-Heep et al., 1983; Möller et al., 1987; Thauer et al., 1989).

A widespread modification of the oxidative TCA cycle features a CoA-transferase, which catalyzes the conversion of succinyl-CoA into succinate along with the activation of acetate to acetyl-CoA. This strategy is particularly advantageous for bacteria that grow on acetate because it permits the saving of one ATP equivalent for the activation of acetate. Indeed, the conversion of acetate to acetyl-CoA is usually catalyzed by acetyl-CoA synthetase that forms AMP and PP_i_ from ATP. Considering the following hydrolysis of PP_i_ into two phosphate molecules, two ATPs have to be spent for the formation of one acetyl-CoA. On the contrary, employing a CoA-transferase allows to activate acetate at the expense of only one ATP (i.e., ATP that is not produced by the succinyl-CoA synthetase reaction), with one enzyme performing both processes:


acetate+succinyl-CoA→acetyl-CoA+succinate

⁢vs.

$$\text{succinyl - CoA}+\text{ADP}+{\text{P}}_{\text{i}}\to \text{succinate}+\text{CoA}+\text{ATP}$$

$$\text{acetate}+\text{CoA}+\text{ATP}\to \text{acetyl}-\text{CoA}+\text{AMP}+\text{PP}{}_{\text{i}}$$

AMP+ATP→2⁢ADP

$${\text{PP}}_{\text{i}}\to 2{\text{P}}_{\text{i}}$$

This variant of the TCA cycle was first discovered in the anaerobic sulfate reducers *Desulfobacter postgatei* and *D. hydrogenophilus* and in the sulfur reducer *Desulfuromonas acetoxidans* (Brandis-Heep et al., 1983; Gebhardt et al., 1985; Schauder et al., 1987; Thauer et al., 1989; Schmitz et al., 1990). Later, this pathway was also shown in the aerobic *Acetobacterium aceti* and in the microaerophilic *Snodgrassella alvi* that grow at high acetate concentrations and thus do not require an expensive mechanism of acetate activation (Mullins et al., 2008; Kwong et al., 2017). It probably functions in *Geobacter sulfurreducens* (Deltaproteobacteria) and *Limisalsivibrio acetivorans* (Deferribacterota) that also use the TCA cycle for anaerobic acetate oxidation (Segura et al., 2008; Mollaei et al., 2021; Spring et al., 2022). In contrast, microorganisms using the acetyl-CoA synthase/carbon monoxide dehydrogenase pathway for acetate oxidation do not produce succinyl-CoA in their catabolism and activate acetate with acetate kinase and phosphotransacetylase at the expense of one ATP equivalent (Thauer et al., 1989). It can be concluded that strict anaerobes operating the TCA cycle for acetate oxidation take advantage of CoA-transferases for the activation of acetate, a strategy that allows them to save ATP and deal better with the energy limitations of their metabolism. The only organism that appears to break this rule is the sulfur reducer *Desulfurella acetivorans* that activates acetate *via* the acetate kinase/phosphotransacetylase pathway but oxidizes acetate *via* the TCA cycle (Schmitz et al., 1990). Nevertheless, *D. acetivorans* possesses four CoA-transferase homologs in the genome, and succinyl-CoA:acetate CoA-transferase activity was later measured in *D. acetivorans* cell extracts (Mall et al., 2018). During our investigation of *D. acetivorans* propionate metabolism, we noticed that the CoA-transferase encoded by AHF96498 gene was one of the most strongly down-regulated proteins in propionate- (compared to acetate-) grown cells, while succinyl-CoA:acetate CoA-transferase activity followed the same trend. These data suggested that this proteins was responsible for the succinyl-CoA:acetate CoA-transferase reaction *in vivo*. Therefore, we decided to re-investigate the mechanism of acetate activation in this bacterium. Here we characterize a CoA-transferase encoded by the AHF96498 gene, show that it functions in *D. acetivorans* for acetate activation, and discuss the distribution, phylogeny and evolution of this variant of the oxidative TCA cycle.

## Materials and methods

### Materials and equipment

Chemicals and biochemicals were obtained from Sigma-Aldrich, Merck, Roth, VWR, or AppliChem. Materials for molecular biology were purchased from New England BioLabs. Materials and equipment for protein purification were obtained from GE Healthcare, Macherey-Nagel or Millipore. Lead acetate paper was obtained from Macherey-Nagel. Primers were synthesized by Sigma-Aldrich.

### Microbial strains and growth conditions

*Desulfurella acetivorans* A63 (DSM 5264) was obtained from the Deutsche Sammlung von Mikroorganismen und Zellkulturen (DSMZ). The cells were grown in medium containing 6.2 mM NH_4_Cl, 2.2 mM CaCl_2_⋅2H_2_O, 1.6 mM MgCl_2_⋅6H_2_O, 4.4 mM KCl, 1.7 mM KH_2_PO_4_, 1.3 mM K_2_HPO_4_, 10 g l^–1^ sulfur powder, 1 ml l^–1^ SL-10 trace element solution (3 g l^–1^ FeCl_2_⋅4H_2_O, 70 mg l^–1^ ZnCl_2_, 100 mg l^–1^ MnCl_2_⋅4H_2_O, 4 mg l^–1^ CuCl_2_⋅2H_2_O, 24 mg l^–1^ NiCl_2_⋅6H_2_O, 36 mg l^–1^ Na_2_MoO_4_⋅2H_2_O, 30 mg l^–1^ H_3_BO_3_, 224 mg l^–1^ CoCl_2_⋅6H_2_O) and 1 ml l^–1^ Wolfe’s vitamin solution (20 mg l^–1^ biotin, 20 mg l^–1^ folic acid, 100 mg l^–1^ pyridoxamine dihydrochloride, 50 mg l^–1^ thiamine dihydrochloride, 50 mg l^–1^ riboflavin, 50 mg l^–1^ nicotinic acid, 50 mg l^–1^ DL-Ca-pantothenate, 1 mg l^–1^ cyanocobalamin, 50 mg l^–1^ 4-aminobenzoic acid and 50 mg l^–1^ lipoic acid). The medium was prepared without sulfur and vitamin solution, made anaerobic by bubbling with N_2_ (100%) and dispensed anaerobically into serum bottles containing sulfur powder. The bottles were sealed with butyl rubber stoppers and aluminum caps and autoclaved for 40 min at 110°C. Before inoculation, the medium was reduced by the addition of Na_2_S⋅9H_2_O to a final concentration of 0.05% (w/v) and supplemented with vitamins. When cultivated heterotrophically, acetate or propionate was supplemented as sole carbon source to a final concentration of 0.5 and 0.05%, respectively. The gas phase was replaced with N_2_/CO_2_ (80:20; heterotrophic growth) or H_2_/CO_2_ (80:20; autotrophic growth) at 1 bar overpressure. The pH was adjusted with bicarbonate to the strain optimum of 6.8–7.0. Cultures were incubated at 55°C while shaking at 130 rpm. Growth was determined using Neubauer counting chambers.

*Escherichia coli* strains [Top10, Rosetta 2 (DE3)] were grown at 37°C in lysogeny broth medium. Antibiotics were added to the cultures to a final concentration of 100 μg ml^–1^ ampicillin and 34 μg ml^–1^ chloramphenicol.

### *Desulfurella acetivorans* cell extract preparation

Cultures (200 ml) were filtrated aerobically (Whatman GF/D, 47 mm) to remove the elemental sulfur before centrifugation (15,000 × *g*, 4°C, 20 min). The supernatant was discarded gently and the resulting cell pellet was resuspended with 20 mL of fresh sulfur-free medium. The cell suspension was transferred to a 50 mL centrifuge tube and centrifuged again (21,000 × *g*, 4°C, 20 min). After having discarded the supernatant, the cells were frozen with liquid nitrogen and stored at −80°C for up to 6 months. To perform enzymatic assays, the frozen cells were thawed on ice and resuspended in 20 mM Tris–HCl (pH 7.8 at 55°C), 5 mM dithiothreitol (DTT), and lysed on ice with the Sonopuls ultrasonic homogenizer (BANDELIN Electronic GmbH; 60% amplitude, 4 min, 1-s pulse, 2-s breaks; total energy input 2,000 kJ). The insoluble cell debris was removed by centrifugation (21,000 × *g*, 4°C, 20 min).

### CoA-esters synthesis

Acetyl-CoA, propionyl-CoA and succinyl-CoA were synthesized from the corresponding anhydrides and CoA according to Simon and Shemin (1953).

### Cloning of *Desulfurella acetivorans* CoA-transferase encoded by the AHF96498 gene in *Escherichia coli*

Standard protocols were used for the purification, cloning, transformation and amplification of DNA (Ausubel et al., 1987). The AHF96498 encoding gene was amplified by PCR with Q5 High-Fidelity DNA Polymerase using a forward primer (5′-ATAT**GAATTC**ATGGGTCACGGAAAAG-3′) introducing an *Eco*RI site (bold) and a reverse primer (5′-TAAT**CTCGAG**TTAAAAGGCCAGCTTC-3′) introducing an *Xho*I site (bold). PCR conditions were as follows: 30 cycles of 30 s denaturation at 98°C, 60 s primer annealing at 64°C, and 30 s elongation at 72°C. The isolated PCR product was treated with the corresponding restrictases and ligated into the expression vector pET23b containing a sequence encoding a C-terminal His_6_-tag. The plasmid was transformed into *E. coli* TOP10 for amplification, followed by purification and sequencing.

### Heterologous expression in *Escherichia coli*

The amplified expression vector was used to transform *E. coli* Rosetta 2 (DE3). The cells were grown at 37°C in lysogeny broth medium with ampicillin and chloramphenicol. Expression was induced at an optical density (OD_600_ nm) of 0.5–0.8 with 1 mM isopropyl-ß-D-thiogalactopyranoside (IPTG), and the temperature was lowered to 20°C. The cells were harvested after overnight growth and stored at −20°C until use.

### Preparation of *Escherichia coli* cell extracts

Frozen cells were suspended in a triple volume of 20 mM Tris–HCl (pH 7.8), containing 20 mM NaCl and 0.1 mg ml^–1^ DNase I. The cell suspensions were lysed by a threefold passage through a chilled French pressure cell (103 MPa). To remove thermally unstable *E. coli* proteins, the supernatant (cell extract) has been subjected to a heat precipitation step (80°C; 15 min). The resulting cell lysate was centrifuged (100,000 × *g*; 4°C; 60 min), filtered and used for protein purification.

### Purification of recombinant AHF96498 gene product

The heterologously produced His-tagged protein encoded by the AHF96498 gene was purified by affinity chromatography using a gravity flow separation column (Econo-column, 1.0 cm × 30 cm glass chromatography column, Bio-Rad) loaded with 2-mL Protino Ni-NTA agarose matrix (Macherey-Nagel). The column was equilibrated with 20 mM Tris–HCl (pH 7.8) containing 20 mM NaCl. Cell extract was applied to the column and incubated for 15 min at 4°C. To elute unwanted proteins, the column was washed first with the same equilibration buffer, then two times with the same buffer containing 10 mM imidazole. The recombinant enzyme was eluted with the same buffer containing 500 mM imidazole. The enzyme was concentrated using a 10 K Vivaspin Turbo 4 and stored at −20°C with glycerol (50%, v/v). The identity of the purified recombinant protein was confirmed at the IZKF Core Unit Proteomics Münster with tryptic in-gel digestion and mass spectrometric analysis using Synapt G2 Si coupled to M-Class (Waters Corp, Eschborn, Germany).

### Enzyme assays

CoA-transferase activity of the purified AHF96498 gene product and in *D. acetivorans* cell extracts was measured using ultra high performance liquid chromatography (UHPLC). All the activities were measured at 55°C and the reaction mixtures (40 μl) contained 100 mM Tris–HCl (pH 7.8), 5 mM MgCl_2_, 5 mM DTT, and purified enzyme or cell extract. Activities were tested with acetyl-CoA, succinyl-CoA or propionyl-CoA toward acetate, succinate or propionate. In addition, the purified CoA-transferase was tested also with acetyl-CoA toward butyrate, 4-hydroxybutyrate, formate, methylsuccinate, DL-malate, and glutarate. The reaction was stopped after 1 min by the addition of 20 μl of 1 M HCl/10% acetonitrile (sample:stop solution, 1:1 [v/v]). The specific activities were calculated by considering the peaks of consumed and formed CoA-esters. The concentration of the latter was calculated by multiplying the starting substrate concentration by the relative abundance [%] of the formed CoA-ester integrated peak area.

Succinyl-CoA synthetase activity was measured using UHPLC as the CoA-dependent formation of succinyl-CoA from succinate and CoA. The assay mixture contained 100 mM Tris–HCl (pH 7.8), 5 mM DTT, 5 mM MgCl_2_, 20 mM succinate, 5 mM ATP, 1 mM CoA, and cell extract.

Acetate kinase activity was measured spectrophotometrically following acetate-dependent oxidation of NADH at 365 nm in an assay containing (in 0.3 ml) 100 mM Tris–HCl (pH 7.8), 5 mM DTT, 5 mM MgCl_2_, 20 mM acetate, 5 mM ATP, 0.5 mM NADH, 5 mM PEP, 5 μ pyruvate kinase (rabbit muscle, Sigma P9136), 25 μ lactate dehydrogenase (rabbit muscle, Sigma L2500), and cell extract. The activity of AMP-forming acetyl-CoA synthetase was tested by including 5 μ myokinase and 0.5 mM CoA in the same reaction mixture.

Phosphotransacetylase activity was measured spectrophotometrically in an assay coupled with endogenous citrate synthase and malate dehydrogenase following NAD reduction at 365 nm as the CoA- and acetyl-phosphate dependent acetyl-CoA formation in the reaction mixture containing 100 mM Tris–HCl (7.8), 5 mM DTT, 5 mM NAD, 10 mM malate, 10 mM acetyl-phosphate, 1 mM CoA and cell extract.

### Inactivation experiment

For inactivation experiments, the purified CoA-transferase (20 μg) was treated with NaBH_4_. The enzyme was preincubated with 1 mM acetyl-CoA for 10 min at 55°C, while an assay without acetyl-CoA was used as a control. NaBH_4_ in 1 M NaOH was added to both reactions at a final concentration of 10 mM, followed by the addition of HCl to a final concentration of 10 mM (Friedmann et al., 2006). The reactions were further incubated for 10 min. Finally, 2 μg of the enzyme was used for the measurement of acetyl-CoA:propionate CoA-transferase activity.

### Analytical ultra high performance liquid chromatography

CoA and CoA-esters were detected with Agilent 1290 Infinity II UHPLC using a reversed-phase C18 column (Agilent InfinityLab Poroshell 120 EC-C18 1.9 μm 2.1 mm × 50 mm column). The following acetonitrile gradient in 10 mM potassium phosphate buffer (pH 7) with a flow rate of 0.55 ml min^–1^, was used: from 2 to 8% at 0–2.66 min; from 8 to 30% at 2.66–3.33 min; from 30 to 2% at 3.33–3.68 min; 2% at 3.68–5 min. Retention times were: succinyl-CoA, 0.7 min; CoA, 0.9 min; acetyl-CoA, 1.7 min; propionyl-CoA, 2.4 min; butyryl-CoA, 3.4 min. Reaction products and standard compounds were detected by UV absorbance at 260 nm with a 1290 Infinity II diode array detector (Agilent) and the amount of product was calculated from the relative peak area. The identification of the CoA esters was based on co-chromatography with standards and analysis of the UV spectra of the products.

### Database search and phylogenetic analysis

Query sequences for the database searches were obtained from the NCBI database. For the alignment, we took the sequences of experimentally characterized CoA-transferases used by Hackmann (2021) (96 sequences) in his analysis of CoA-transferases and supplemented them with sequences from *Pseudomonas aeruginosa* (Sasikaran et al., 2014), *Paraburkholderia xenovorans* (Kronen et al., 2015), from Desulfurellaceae species (17 sequences), from a closed genome of a sulfate-reducing bacterium (1 sequence) and from sulfate- and sulfur-reducing bacteria where the CoA-transferase variant of the oxidative TCA cycle was shown experimentally (7 sequences) (Thauer et al., 1989; Segura et al., 2008; Mollaei et al., 2021). The genomes of sulfate reducers selected were at the “Finished” status. Only for the family Desulfurellaceae, all the genomes available from the NCBI database have been considered. The BLASTP searches (Altschul et al., 1990) were performed *via* the NCBI BLAST server^1^ and *via* the Integrated Microbial Genomes and Microbiomes system.^2^ The phylogenetic tree was constructed by using the maximum likelihood method and Jones-Taylor-Thornton (JTT) matrix-based model (Jones et al., 1992) in MEGA11 (Tamura et al., 2021). Altogether, 123 amino acid sequences were used in this analysis. In addition, the same data set was used for the construction of a tree with the maximum-parsimony statistical method and the Subtree-Pruning-Regrafting (SPR) search method.

### Other methods

DNA sequence determination of purified plasmids was performed by Eurofins (Ebersberg, Germany). Protein concentration was measured according to the Bradford method (Bradford, 1976) using BSA as a standard. Sodium dodecyl sulfate-polyacrylamide gel electrophoresis (SDS-PAGE; 12.5%) was performed as previously described (Laemmli, 1970). Proteins were visualized using Coomassie blue staining (Zehr et al., 1989). *K*_*m*_ and *V*_*max*_ values were calculated by GraphPad Prism 5 software.

## Results

### Enzyme activities in *Desulfurella acetivorans* cell extracts

*Desulfurella acetivorans* was described as a thermophilic sulfur-reducing bacterium oxidizing acetate through the TCA cycle, while no significant growth was shown with propionate (Bonch-Osmolovskaya et al., 1990; Schmitz et al., 1990). Later, the capability of this species to grow autotrophically with molecular hydrogen was also demonstrated (Pradella et al., 1998). In our hands, this bacterium could grow autotrophically as well as heterotrophically on acetate or propionate. Acetate-grown cells had only very low activity of succinyl-CoA synthetase, whereas this activity in propionate- and autotrophically grown cells was much higher (Table 1). In contrast, acetate-grown cells showed high activity of succinyl-CoA:acetate CoA-transferase, suggesting that acetate activation in *D. acetivorans* proceeds through the CoA-transfer from succinyl-CoA. The activity of this enzyme in autotrophically- and propionate-grown cells was 4 and 34 times lower than in acetate-grown cells, respectively (Table 1). The participation of a CoA-transferase in acetate activation is further supported by the low activity of enzymes involved in alternative acetate activation pathways. Indeed, the acetate kinase activity was 4 times lower than the succinyl-CoA:acetate CoA-transferase activity (Table 1). Since the addition of myokinase to the reaction mixture to detect AMP formation did not result in any detectable difference in the spectrophotometric assays, the activity of the AMP-forming acetyl-CoA synthetase was absent in acetate-grown *D. acetivorans* cells (or was below the detection limit of 0.02 μmoles min^−1^ mg^–1^ protein). Our comparison of proteomes of acetate- and propionate-grown cells revealed that one of the four CoA-transferases present in *D. acetivorans* genome (AHF96498) was 50-fold down-regulated (Pettinato, König and Berg, manuscript in preparation). Therefore, we decided to characterize this putative succinyl-CoA:acetate CoA-transferase biochemically.

**TABLE 1:** Enzyme activity in extracts of *Desulfurella acetivorans* cells grown under autotrophic or heterotrophic conditions in the presence of acetate or propionate.

Enzyme	Specific activity (in μmoles min^−1^ mg^–1^ protein) in cells grown on		
	
	Acetate	Propionate	CO_2_ + H_2_
Succinyl-CoA:acetate CoA-transferase^a^	0.24 ± 0.08 (*n* = 7)	0.007 ± 0.003 (*n* = 3)	0.06 ± 0.03 (*n* = 5)
Acetyl-CoA:succinate CoA-transferase^a^	0.96 ± 0.11 (*n* = 5)	0.14 ± 0.03 (*n* = 4)	0.6 ± 0.19 (*n* = 3)
Propionyl-CoA:acetate CoA-transferase^a^	16 ± 5.5 (*n* = 4)	0.44 ± 0.12 (*n* = 4)	3.6 ± 0.66 (*n* = 3)
Acetyl-CoA:propionate CoA-transferase^a^	20.1 ± 2.05 (*n* = 6)	1.1 ± 0.18 (*n* = 4)	5.9 ± 1.15 (*n* = 3)
Propionyl-CoA:succinate CoA-transferase^a^	1.05 ± 0.31 (*n* = 5)	0.07 ± 0.03 (*n* = 4)	0.19 ± 0.07 (*n* = 5)
Succinyl-CoA:propionate CoA-transferase^a^	0.23 ± 0.07 (*n* = 5)	0.02 ± 0.01 (*n* = 3)	0.07 ± 0.04 (*n* = 5)
Succinyl-CoA synthetase	0.006 ± 0.006 (*n* = 4)	0.08 ± 0.04 (*n* = 4)	0.02 ± 0.02 (*n* = 3)
Acetate kinase	0.06 ± 0.04 (*n* = 3)	0.02 ± 0.01 (*n* = 3)	0.08 ± 0.02 (*n* = 3)
Phosphotransacetylase	0.72 ± 0.16 (*n* = 3)	0.21 ± 0.04 (*n* = 4)	0.31 ± 0.04 (*n* = 4)

Specific activities were measured at 55°C; data are mean ± s.d. and the number of biological repetitions (*n*) is shown. For each biological replication, at least two technical replications were carried out.

^a^The concentration of CoA esters and carbonic acids in the reaction mixture was 1 and 20 mM, respectively.

### Characterization of the CoA-transferase encoded by AHF96498 gene

**FIGURE 2:**
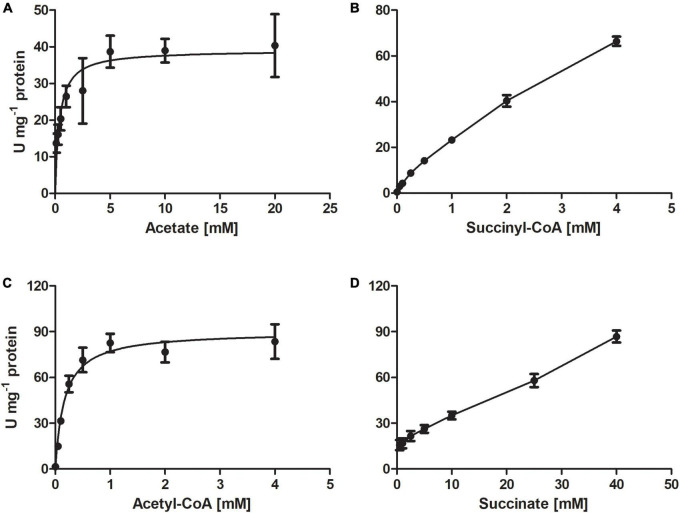
Kinetics of the CoA-transferase encoded by the AHF96498 gene, measured for the acetate:succinyl-CoA **(A,B)** and acetyl-CoA:succinate **(C,D)** CoA-transferase reactions. Specific activities were measured at 55°C; data are mean ± s.d.

The purified recombinant AHF96498 gene product catalyzed CoA-transferase reaction. We examined the ability of this enzyme to utilize different CoA-donors (succinyl-CoA, acetyl-CoA and propionyl-CoA) and CoA-acceptors (acetate, succinate, propionate, butyrate, formate, 4-hydroxybutyrate, methylsuccinate, glutarate, DL-malate). The enzyme was active with all three tested CoA-esters and showed the highest activity with propionate and acetate, lower activity with 4-hydroxybutyrate and butyrate and (almost) no activity with formate, methylsuccinate, malate and glutarate (Table 2). The activity with succinyl-CoA and acetate did not follow a Michaelis-Menten kinetic (Figure 2) but increased linearly with the rise of succinate/succinyl-CoA concentrations (40 μmoles min^−1^ mg^–1^ protein at 2 mM succinyl-CoA and 20 mM acetate, Table 2). The high affinities of the enzyme for acetate/acetyl-CoA (*K*_m_ of 0.4/0.2 mM, respectively) further strengthened our suggestion that its main physiological function is acetate activation and succinate formation in the course of the oxidative TCA cycle. However, it could also use propionyl-CoA as a CoA-donor. Moreover, the highest activity of the enzyme was detected with propionate and acetyl-CoA or acetate and propionyl-CoA as substrates (Table 2). This activity could also be measured in *D. acetivorans* cell extracts and it showed the same trend in autotrophically vs. acetate-/propionate-grown cells as with succinyl-CoA and acetate (Tables 1, 3). Nevertheless, acetyl-CoA:propionate (or propionyl-CoA:acetate) CoA-transferase activity does not have any physiological sense in autotrophic or acetate-grown cells due to the unavailability of propionate/propionyl-CoA and can be explained by the promiscuity of the AHF96498 gene product. Importantly, the *K*_m_ values of the heterologously produced enzyme to the substrates were comparable to the apparent *K*_m_ values in cell extracts, further confirming the major role of this enzyme in the *D. acetivorans* TCA cycle (compare Tables 2, 3). To understand the reaction mechanism of the protein, an inactivation test with NaBH_4_ has been performed. The reduction of a glutamyl-CoA intermediate by borohydride would lead to the consequent loss of activity by inactivation of the catalytic glutamate residue, which is typical for class I CoA-transferases (Solomon and Jencks, 1968; Tung and Wood, 1975; Moore and Jencks, 1982). The CoA-free enzyme did not show any reduction of activity, whereas the enzyme pre-incubated with acetyl-CoA and subsequently treated with borohydride retained only 4.5% of the original activity, thus identifying AHF96498 as a class I CoA-transferase.

**TABLE 2:** Catalytic properties of the heterologously produced succinyl-CoA:acetate CoA-transferase encoded by the AHF96498 gene.

First substrate (mM)	Second substrate (mM)	*V*_max_ or specific activity (in μmoles min^−1^ mg^–1^ protein)	*K*_m_ (in mM)	*k*_cat_/*K*_m_ (in s^–1^ mM^–1^)
Succinyl-CoA (0.005 to 4)	Acetate (20)	40 ± 2.5 (at 2 mM)^a^	NA	NA
Acetate (0.1 to 20)	Succinyl-CoA (2)	9 ± 0.3	0.4 ± 0.07	23
Acetyl-CoA (0.005 to 4)	Succinate (40)	90 ± 4.8	0.2 ± 0.04	534
Succinate (0.5 to 40)	Acetyl-CoA (2)	35 ± 2.6 (at 10 mM)^a^	NA	NA
Acetyl-CoA (0.005 to 4)	Propionate (20)	1051 ± 38	0.6 ± 0.06	1867
Propionate (0.1 to 20)	Acetyl-CoA (2)	875 ± 59	3.9 ± 0.8	223
Propionyl-CoA (0.005 to 4)	Acetate (20)	669 ± 76	1.1 ± 0.3	584
Acetate (0.1 to 10)	Propionyl-CoA (2)	766 ± 46	1 ± 0.2	749
Propionyl-CoA (0.005 to 4)	Succinate (20)	56 ± 4.1	0.3 ± 0.09	171
Succinate (0.5 to 40)	Propionyl-CoA (2)	29 ± 3 (at 10 mM)^a^	NA	NA
Succinyl-CoA (0.005 to 2)	Propionate (20)	17 ± 1.7	1.2 ± 0.3	14
Propionate (0.1 to 20)	Succinyl-CoA (2)	8.5 ± 0.2	0.3 ± 0.03	33
4-Hydroxybutyrate (0.25 to 50)	Acetyl-CoA (1)	7.7 ± 0.3	0.4 ± 0.1	24
Butyrate (0.1 to 80)	Acetyl-CoA (1)	92 ± 8.3	17 ± 4	5
Formate (50 to 1000)	Acetyl-CoA (1)	74 ± 11	666 ± 176	0.1
Methylsuccinate (10 to 100)	Acetyl-CoA (1)	1 ± 0.3 (at 10 mM)^a^	NA	NA
Methylsuccinate (2.5 to 50)	Propionyl-CoA (1)	35 ± 0.8 (at 10 mM)^a^	NA	NA
DL-malate (20)	Acetyl-CoA (1)	0.1 ± 0.01	NA	NA
Glutarate (20)	Acetyl-CoA (1)	0.1 ± 0.02	NA	NA

Specific activities were measured at 55°C; data are mean ± s.d.

NA, not applicable.

^a^Non-Michaelis-Menten kinetics, specific activity of the enzyme is shown.

**TABLE 3:** Kinetics of CoA-transferase reaction in extracts of *Desulfurella acetivorans* cells grown under different conditions.

First substrate (mM)	Second substrate (mM)	Acetate-grown		Propionate-grown		Autotrophic	
				
		*V*_*max*_ or specific activity (in μmoles min^−1^ mg^–1^ protein)	*K*_*m*_ (in mM)	*V*_*max*_ or specific activity (in μmoles min^−1^ mg^–1^ protein)	*K*_*m*_ (in mM)	*V*_*max*_ or specific activity (in μmoles min^−1^ mg^–1^ protein)	*K*_*m*_ (in mM)
Acetyl-CoA (0.005 to 2)	Propionate (20)	42 ± 5.5	0.9 ± 0.3	1.5 ± 0.1	0.2 ± 0.04	5.2 ± 0.4	0.2 ± 0.06
Propionate (0.1 to 20)	Acetyl-CoA (2)	20 ± 1.6	1.7 ± 0.5	1.4 ± 0.05	0.4 ± 0.06	4.6 ± 0.3	0.7 ± 0.2
Acetyl-CoA (0.005 to 2)	Succinate (20)	0.5 ± 0.1	0.06 ± 0.06	0.2 ± 0.02	0.04 ± 0.03	0.4 ± 0.03	0.02 ± 0.02
Succinate (0.5 to 40)	Acetyl-CoA (2)	0.09 ± 0.01 (at 10 mM)^a^	NA	0.5 ± 0.4 (at 10 mM)^a^	NA	0.2 ± 0.01 (at 10 mM)^a^	NA

Please note that apparent *V_max_* and *K_m_* values are shown.

Specific activities were measured at 55°C; data are mean ± s.d., at least two technical replications were carried out. NA, not applicable.

^a^Non-Michaelis-Menten kinetics, specific activity of the enzyme is shown.

### Phylogenetic analysis

**FIGURE 3:**
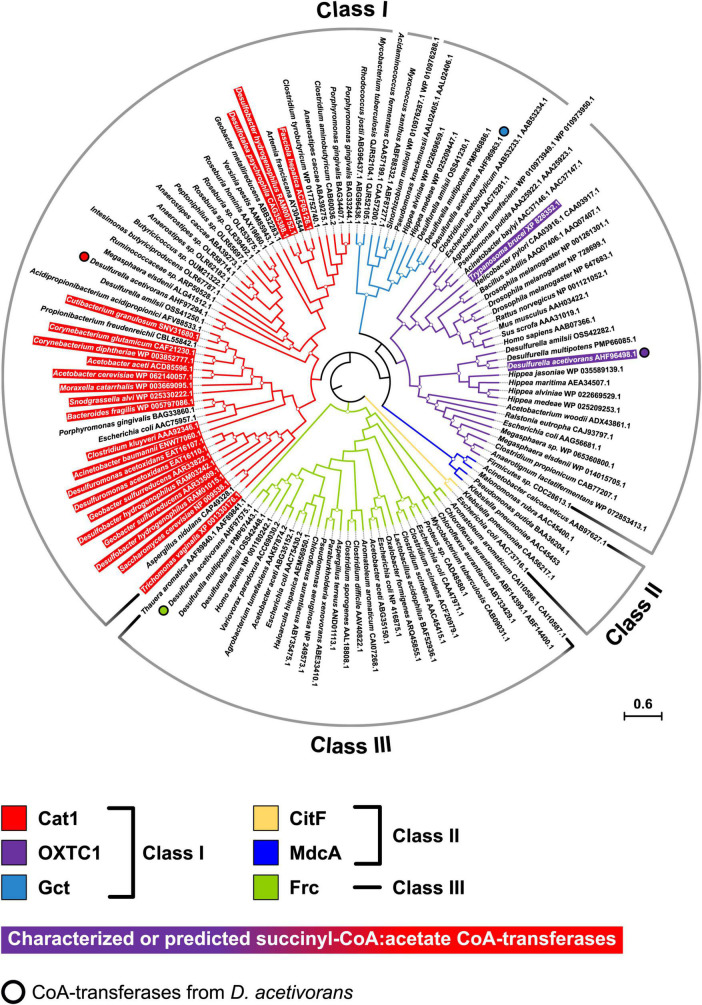
Phylogenetic tree of CoA-transferases constructed by maximum likelihood algorithm. The scale bar represents a difference of 0.6 amino acid substitutions per site. The percentage bootstrap values for the clade of this group were calculated in 500 replications. The clades with bootstrap values above 70% are marked with circles. The tree constructed by maximum-parsimony algorithm was similar with minor exceptions (not shown).

To better understand the evolution of *D. acetivorans* CoA-transferases, we performed the phylogenetic analysis of CoA-transferases belonging to different families. The resulting phylogenetic tree (Figure 3) was similar to the tree published by Hackmann (2021), where a new six-family classification of CoA-transferases was proposed, elaborating the previous three-family classification of Heider (2001). The characterized succinyl-CoA:acetate CoA-transferase encoded by the AHF96498 gene belonged to the OXTC1/family I CoA-transferases; close homologs of this protein were found in all sequenced Desulfurellaceae, suggesting that they all use the CoA-transferase variant of the TCA cycle. Interestingly, the CoA-transferases of species in which this variant of the TCA cycle was discovered (i.e., *Desulfuromonas acetoxidans* and *Desulfobacter hydrogenophilus*) belonged to another family (Cat1/family I; Figure 3). Notably, most of the Cat1 proteins show a preference for short acyl-CoAs (C_2_–C_4_), with 13 of them being already characterized as succinyl-CoA:acetate CoA-transferases (Hackmann, 2021). The phylogenetic analysis also revealed that two other CoA-transferases from *D. acetivorans*, AHF97294, and AHF97575, belonged to the Cat1/family I and the Frc/family III CoA-transferases, respectively, whereas the CoA-transferase AHF96963 belonged to the Gct/family I cluster (Figure 3).

## Discussion

In this study we showed that *D. acetivorans* uses a succinyl-CoA:acetate CoA-transferase for acetate activation with the concomitant conversion of succinyl-CoA to succinate, similarly to other strictly anaerobic bacteria that oxidize acetate using the TCA cycle (Thauer et al., 1989). Due to the low activities of acetate kinase and succinyl-CoA synthetase, the previously proposed variant of acetate activation (Schmitz et al., 1990) (i.e., *via* acetyl phosphate with succinyl-CoA synthetase responsible for succinyl-CoA conversion to succinate) is of only minor importance for acetate oxidation in *D. acetivorans*. On the contrary, the acetate kinase/phosphotransacetylase pathway appears to be specific for microorganisms that oxidize acetyl-CoA *via* the carbon monoxide dehydrogenase/acetyl-CoA synthase pathway (Thauer et al., 1989). Indeed, these microorganisms either do not form succinyl-CoA in their metabolism or operate succinyl-CoA synthetase mainly in the other direction (i.e., succinyl-CoA formation followed by carboxylation in the 2-oxoglutarate synthase reaction), making the usage of a CoA-transferase disadvantageous. Please note that a CoA-transferase cannot represent the only acetate activation mechanism in *D. acetivorans*. Indeed, the amount of acetate activated through this pathway is equivalent to the amount of succinyl-CoA converted into succinate in the TCA cycle, whereas *D. acetivorans* requires additional acetyl-CoA for biosynthetic reactions. This additional acetyl-CoA has to be produced *via* the acetate kinase and phosphotransacetylase reactions.

Although our data indicate that the AHF96498 gene product functions as a succinyl-CoA:acetate CoA-transferase under physiological conditions, this is not the only CoA-transferase functioning in *D. acetivorans*. This is also evident from the presence in cell extracts of, e.g., propionyl-CoA:acetate CoA-transferase activity under conditions when the AHF96498 gene is down-regulated (Table 1). Which of the three other CoA-transferases can be responsible for this (and other) activities, and what are the physiological functions of these enzymes, is a subject of further investigations.

All sequenced representatives of Desulfurellaceae possessed a close homolog of the AHF96498-CoA-transferase (≥82/91% amino acid identity/similarity; Figure 3), suggesting that all members of this family use the CoA-transferase variant of the TCA cycle. In *Hippea maritima* and *H. jasoniae*, this enzyme was the only CoA-transferase identified in the genome (Figure 3); the corresponding protein was present in the proteome of mixotrophically grown *H. maritima* cells (Steffens et al., 2021). Furthermore, our findings allowed a re-interpretation of the results of the omics analysis of *D. amilsii* metabolism (Florentino et al., 2017, 2019). Based on the early data on *D. acetivorans* metabolism (Schmitz et al., 1990), the possibility of the functioning in *D. amilsii* of a CoA-transferase variant of the TCA cycle was neglected, and the participation of acetyl-CoA synthetase in acetate activation was proposed (Florentino et al., 2017, 2019). However, a CoA-transferase homologous to the AHF96498 gene product was also present in *D. amilsii* (DESAMIL20_1835, 95/98% of identity/similarity) and was much more abundant in the proteome of acetate-grown cells than the putative acetyl-CoA synthetase DESAMIL20_135 (PXD008496; Florentino et al., 2019), further confirming its participation in acetate oxidation.

A possible drawback of the usage of the succinyl-CoA:acetate CoA-transferase for acetate activation is its slightly unfavorable equilibrium for acetyl-CoA formation (*K*_*eq*_ = 0.14; Mullins et al., 2008). However, this problem is not relevant for *D. acetivorans*, since its citrate synthase has low *K*_*m*_ value and extremely high activity in cell extracts (Mall et al., 2018; Steffens et al., 2021), shifting the equilibrium of the CoA-transferase reaction to the products. In addition, acetate concentrations are typically high in many anaerobic ecosystems, further moving the equilibrium toward products formation. In contrast, aerobes using AMP-forming acetyl-CoA synthetase usually deal with much lower acetate availability and thus need to invest additional energy in its activation. All in all, the CoA-transferase variant of the oxidative TCA cycle appears to be widespread in (and optimal for) anaerobic bacteria oxidizing acetate *via* the TCA cycle.

The functioning of the CoA-transferase variant of the TCA cycle in Desulfurellaceae explains the similarities in the carbon isotope effects associated with the oxidation of acetate during growth of various sulfur reducers (Goevert and Conrad, 2010). Indeed, the differences in fractionation between sulfur reducers using the CoA-transferase/citrate synthase version of the TCA cycle are within 5 ‰ differences for bacteria using the same biochemical pathway for the oxidation of acetate (−6.3 and −8.4 ‰ in *D. acetivorans* and *H. maritima* vs. −11.5 and −11.2 ‰ in *Desulfuromonas acetoxidans* and *D. thiophila*, respectively; Goevert and Conrad, 2010). These data confirm the initial interpretation that carbon isotope fractionation of acetate depends on the metabolic pathway used for the acetate oxidation (i.e., acetyl-CoA/CO dehydrogenase pathway, ATP-citrate-lyase-TCA cycle or citrate-synthase-TCA-cycle) (Goevert and Conrad, 2008).

Our phylogenetic analysis revealed that the studied CoA-transferase (AHF96498 gene product) belongs to the OXTC1/Class I family of CoA-transferases that preferably use oxo- and hydroxy-acyl-CoAs as substrates (Hackmann, 2021), while the other succinyl-CoA:acetate CoA-transferases identified so far cluster in a different and distant phylogenetic group, the Cat1/Class I family (Figure 3). These data suggest convergent evolution of this variant of the cycle in different bacterial groups. Interestingly, some eukaryotes (*Trypanosoma brucei* and *Fasciola hepatica*) possess a succinyl-CoA:acetate CoA-transferase that works in the opposite direction (for succinyl-CoA synthesis), being involved in acetate production during anaerobic metabolism and working together with succinyl-CoA synthetase for ATP formation *via* substrate phosphorylation (van Grinsven et al., 2009; Mochizuki et al., 2020). The characterized eukaryotic enzymes also belong to different families (OXTC1 and Cat1, respectively), highlighting the frequent convergent evolution of different metabolic processes that involve CoA-transferases.

## Data availability statement

The original contributions presented in this study are included in the article/supplementary material, further inquiries can be directed to the corresponding author.

## Author contributions

IB designed experiments. EP and PB performed experiments and analyzed the data and wrote the manuscript. All authors read and approved the final manuscript.

## Footnotes

1
http://www.ncbi.nlm.nih.gov/BLAST/

2
https://img.jgi.doe.gov/m/

## References

[B1] Altschul S. F. Gish W. Miller W. Myers E. W. Lipman D. J. (1990). Basic local alignment search tool. *J. Mol. Biol.* 215 403–410. 10.1016/S0022-2836(05)80360-2 2231712

[B2] Aoshima M. Ishii M. Igarashi Y. (2004a). A novel enzyme, citryl-CoA synthetase, catalysing the first step of the citrate cleavage reaction in *Hydrogenobacter thermophilus* TK-6. *Mol. Microbiol.* 52 751–761. 10.1111/j.1365-2958.2004.04009.x 15101981

[B3] Aoshima M. Ishii M. Igarashi Y. (2004b). A novel enzyme, citryl-CoA lyase, catalysing the second step of the citrate cleavage reaction in *Hydrogenobacter thermophilus* TK-6. *Mol. Microbiol.* 52 763–770. 10.1111/j.1365-2958.2004.04010.x 15101982

[B4] Ausubel F. M. Brent R. Kingston R. E. Moore D. D. Seidman J. G. Smith J. A. (1987). *Current protocols in molecular biology.* New York, NY: John Wiley and Sons, 10.1002/mrd.1080010210

[B5] Beck S. Schink B. (1995). Acetate oxidation through a modified citric acid cycle in *Propionibacterium freundenreichii*. *Arch. Microbiol.* 163 182–187. 10.1007/BF00305351

[B6] Berg I. A. (2011). Ecological aspects of the distribution of different autotrophic CO_2_ fixation pathways. *Appl. Environ. Microbiol.* 77 1925–1936. 10.1128/AEM.02473-10 21216907 PMC3067309

[B7] Bonch-Osmolovskaya E. A. Sokolova T. G. Kostrikina N. A. Zavarzin G. A. (1990). *Desulfurella acetivorans* gen. nov. and sp. nov. - a new thermophilic sulfur-reducing eubacterium. *Arch. Microbiol.* 153 151–155. 10.1007/BF00247813

[B8] Bradford M. M. (1976). A rapid and sensitive method for the quantitation of microgram quantities of protein utilizing the principle of protein-dye binding. *Anal. Biochem.* 72 248–254. 10.1016/0003-2697(76)90527-3 942051

[B9] Brandis-Heep A. Gebhardt N. A. Thauer R. K. Widdel F. Pfennig N. (1983). Anaerobic acetate oxidation to CO_2_ by *Desulfobacter postgatei*. *Arch. Microbiol.* 136 222–229. 10.1007/BF00409849

[B10] Buchanan B. B. Sirevåg R. Fuchs G. Ivanovsky R. N. Igarashi Y. Ishii M. (2017). The Arnon-Buchanan cycle: A retrospective, 1966-2016. *Photosynth. Res.* 134 117–131. 10.1007/s11120-017-0429-0 29019085

[B11] Evans M. C. W. Buchanan B. B. Arnon D. I. (1966). A new ferredoxin-dependent carbon reduction cycle in a photosynthetic bacterium. *Proc. Natl. Acad. Sci. U.S.A.* 55 928–934. 10.1073/pnas.55.4.928 5219700 PMC224252

[B12] Florentino A. P. Pereira I. A. C. Boeren S. van den Born M. Stams A. J. M. Sánchez-Andrea I. (2019). Insight into the sulfur metabolism of *Desulfurella amilsii* by differential proteomics. *Environ. Microbiol.* 21 209–225. 10.1111/1462-2920.14442 30307104 PMC6378623

[B13] Florentino A. P. Stams A. J. M. Sánchez-Andrea I. (2017). Genome sequence of *Desulfurella amilsii* strain TR1 and comparative genomics of *Desulfurellaceae* family. *Front. Microbiol.* 8:222. 10.3389/fmicb.2017.00222 28265263 PMC5317093

[B14] Friedmann S. Alber B. E. Fuchs G. (2006). Properties of succinyl-coenzyme A:D-citramalate coenzyme A transferase and its role in the autotrophic 3-hydroxypropionate cycle of *Chloflexus aurantiacus*. *J. Bacteriol.* 188 6460–6468. 10.1128/JB.00659-06 16952935 PMC1595468

[B15] Fuchs G. (2011). Alternative pathways of carbon dioxide fixation: insights into the early evolution of life? *Annu. Rev. Microbiol.* 65 631–658. 10.1146/annurev-micro-090110-102801 21740227

[B16] Gebhardt N. A. Thauer R. K. Linder D. Kaulfers P.-M. Pfennig N. (1985). Mechanism of acetate oxidation to CO_2_ with elemental sulfur in *Desulfuromonas acetoxidans*. *Arch. Microbiol.* 141 392–398. 10.1007/BF00428855

[B17] Goevert D. Conrad R. (2008). Carbon isotope fractionation by sulfate-reducing bacteria using different pathways for the oxidation of acetate. *Environ. Sci. Technol.* 42 7813–7817. 10.1021/es800308z 19031865

[B18] Goevert D. Conrad R. (2010). Stable carbon isotope fractionation by acetotrophic sulfur-reducing bacteria. *FEMS Microbiol. Ecol.* 71 218–225. 10.1111/j.1574-6941.2009.00811.x 20002180

[B19] Hackmann T. J. (2021). Redefining the coenzyme A transferase superfamily with a large set of manually annotated proteins. *Protein Sci.* 31 864–881. 10.1002/pro.4277 35049101 PMC8927868

[B20] Harold L. K. Jinich A. Hards K. Cordeiro A. Keighley L. M. Cross A. (2022). Deciphering functional redundancy and energetics of malate oxidation in mycobacteria. *J. Biol. Chem.* 298:101859. 10.1016/j.jbc.2022.101859 35337802 PMC9062433

[B21] Heider J. (2001). A new family of CoA-transferases. *FEBS Lett.* 509 345–349. 10.1016/s0014-5793(01)03178-7 11749953

[B22] Ivanovsky R. N. Sintsov N. V. Kondratieva E. N. (1980). ATP-linked citrate lyase activity in the green sulfur bacterium *Chlorobium limicola* forma *thiosulfatophilum*. *Arch. Microbiol.* 128 239–241. 10.1007/BF00406165

[B23] Jones D. T. Taylor W. R. Thornton J. M. (1992). The rapid generation of mutation data matrices from protein sequences. *Bioinformatics* 8 275–282. 10.1093/bioinformatics/8.3.275 1633570

[B24] Kerscher L. Oesterhelt D. (1981). Purification and properties of two 2-oxoacid:ferredoxin oxidoreductases from *Halobacterium halobium*. *Eur. J. Biochem.* 116 587–594. 10.1111/j.1432-1033.1981.tb05376.x 6266826

[B25] Kronen M. Sasikaran J. Berg I. A. (2015). Mesaconase activity of class I fumarase contributes to mesaconate utilization by *Burkholderia xenovorans*. *Appl. Environ. Microbiol.* 81 5632–5638. 10.1128/AEM.00822-15 26070669 PMC4510160

[B26] Kwong W. K. Zheng H. Moran N. A. (2017). Convergent evolution of a modified, acetate-driven TCA cycle in bacteria. *Nat. Microbiol.* 2:17067. 10.1038/nmicrobiol.2017.67 28452983 PMC5482284

[B27] Laemmli U. K. (1970). Cleavage of structural proteins during the assembly of the head of bacteriophage T4. *Nature* 227 680–685. 10.1038/227680a0 5432063

[B28] Mall A. Sobotta J. Huber C. Tschirner C. Kowarschik S. Bačnik K. (2018). Reversibility of citrate synthase allows autotrophic growth of a thermophilic bacterium. *Science* 359 563–567. 10.1126/science.aao2410 29420287

[B29] Mochizuki K. Inaoka D. K. Mazet M. Shiba T. Fukuda K. Kurasawa H. (2020). The ASCT/SCS cycle fuels mitochondrial ATP and acetate production in *Trypanosoma brucei*. *Biochim. Biophys. Acta Bioenerg*. 1861 1482–1483. 10.1016/j.bbabio.2020.148283 32763239 PMC7402102

[B30] Molenaar D. van der Rest M. E. Drysch A. Yücel R. (2000). Functions of the membrane-associated and cytoplasmic malate dehydrogenases in the citric acid cycle of *Corynebacterium glutamicum*. *J. Bacteriol.* 182 6884–6891. 10.1128/JB.182.24.6884-6891.2000 11092846 PMC94811

[B31] Mollaei M. Timmers P. H. A. Suarez-Diez M. Boeren S. van Gelder A. H. Stams A. J. M. (2021). Comparative proteomics of *Geobacter sulfurreducens* PCA^T^ in response to acetate, formate and/or hydrogen as electron donor. *Environ. Microbiol.* 23 299–315. 10.1111/1462-2920.15311 33185968 PMC7894505

[B32] Möller D. Schauder R. Fuchs G. Thauer R. K. (1987). Acetate oxidation to CO_2_ via a citric acid cycle involving an ATP-citrate lyase: a mechanism for the synthesis of ATP via substrate level phosphorylation in *Desulfobacter postgatei* growing on acetate and sulfate. *Arch. Microbiol.* 148 202–207. 10.1007/BF00414812

[B33] Moore S. A. Jencks W. P. (1982). Formation of active site thiol esters of CoA transferase and the dependence of catalysis on specific binding interactions. *J. Biol. Chem.* 257 10893–10907. 10.1016/S0021-9258(18)33908-5 6955308

[B34] Mullins E. A. Francois J. A. Kappock T. J. (2008). A specialized citric acid cycle requiring succinyl-coenzyme A (CoA):acetate CoA-transferase (AarC) confers acetic acid resistance on the acidophile. *Acetobacter aceti*. *J. Bacteriol.* 190 4933–4940. 10.1128/JB.00405-08 18502856 PMC2447011

[B35] Nunoura T. Chikaraishi Y. Izaki R. Suwa T. Sato T. Harada T. (2018). A primordial and reversible TCA cycle in a facultatively chemolithoautotrophic thermophile. *Science* 359 559–563. 10.1126/science.aao3407 29420286

[B36] Pradella S. Hippe H. Stackebrandt E. (1998). Macrorestriction analysis of *Desulfurella acetivorans* and *Desulfurella multipotens*. *FEMS Microbiol. Lett.* 159 137–144. 9485604 10.1111/j.1574-6968.1998.tb12852.x

[B37] Sasikaran J. Ziemski M. Zadora P. K. Fleig A. Berg I. A. (2014). Bacterial itaconate degradation promotes pathogenicity. *Nat. Chem. Biol.* 10 371–377. 10.1038/nchembio.1482 24657929

[B38] Schauder R. Widdel F. Fuchs G. (1987). Carbon assimilation pathways in sulfate-reducing bacteria II. Enzymes of a reductive citric acid cycle in the autotrophic *Desulfobacter hydrogenophilus*. *Arch. Microbiol.* 148 218–225.

[B39] Schmitz R. A. Bonch-Osmolovskaya E. A. Thauer R. K. (1990). Different mechanisms of acetate activation in *Desulfurella acetivorans* and *Desulfuromonas acetoxidans*. *Arch. Microbiol.* 154 274–279. 10.1007/BF00248967

[B40] Segura D. Mahadevan R. Juárez K. Lovley D. R. (2008). Computational and experimental analysis of redundancy in the central metabolism of *Geobacter sulfurreducens*. *PLoS Comput. Biol.* 4:e36. 10.1371/journal.pcbi.0040036 18266464 PMC2233667

[B41] Simon E. J. Shemin D. (1953). The preparation of *S*-succinyl coenzyme A. *J. Am. Chem. Soc.* 75:2520.

[B42] Solomon F. Jencks W. P. (1968). Identification of an enzyme-γ-glutamyl coenzyme A intermediate from coenzyme A transferase. *J. Biol. Chem*. 244 1079–1108. 10.1016/S0021-9258(18)91898-3 5769180

[B43] Spring S. Rohde M. Bunk B. Spröer C. Will S. E. Neumann-Schaal M. (2022). New insights into the energy metabolism and taxonomy of Deferribacteres revealed by the characterization of a new isolate from a hypersaline microbial mat. *Environ. Microbiol.* 24 2543–2575. 10.1111/1462-2920.15999 35415868

[B44] Steffens L. Pettinato E. Steiner T. M. Mall A. König S. Eisenreich W. (2021). High CO_2_ levels drive the TCA cycle backwards towards autotrophy. *Nature* 592 784–788. 10.1038/s41586-021-03456-9 33883741

[B45] Tamura K. Stecher G. Kumar S. (2021). MEGA11: Molecular evolutionary genetic analysis version 11. *Mol. Biol. Evol.* 38 3022–3027. 10.1093/molbev/msab120 33892491 PMC8233496

[B46] Thauer R. K. Möller-Zinkhan D. Spormann A. M. (1989). Biochemistry of acetate catabolism in anaerobic chemotrophic bacteria. *Annu. Rev. Microbiol.* 43 43–67. 10.1146/annurev.mi.43.100189.000355 2679359

[B47] Tung K. K. Wood W. A. (1975). Purification, new assay, and properties of coenzyme A transferase from *Peptostreptococcus elsdenii*. *J. Bacteriol*. 124 1462–1474. 10.1128/jb.124.3.1462-1474.1975 369 PMC236061

[B48] van Grinsven K. W. van Hellemond J. J. Tielens A. G. (2009). Acetate:succinate CoA-transferase in the anaerobic mitochondria of *Fasciola hepatica*. *Mol. Biochem. Parasitol* 164 74–79. 10.1016/j.molbiopara.2008.11.008 19103231

[B49] Zehr B. D. Savin T. J. Hall R. E. (1989). A one-step, low background Coomassie staining procedure for polyacrylamide gels. *Anal. Biochem.* 182 157–159. 10.1016/0003-2697(89)90734-3 2481413

[B50] Zhang S. Bryant D. A. (2011). The tricarboxylic acid cycle in cyanobacteria. *Science* 334 1551–1553. 10.1126/science.1210858 22174252

